# AtbZIP62 Acts as a Transcription Repressor to Positively Regulate ABA Responses in Arabidopsis

**DOI:** 10.3390/plants11223037

**Published:** 2022-11-10

**Authors:** Saddam Hussain, Yuxin Cheng, Yingying Li, Wei Wang, Hainan Tian, Na Zhang, Yating Wang, Yuan Yuan, Hadia Hussain, Rao Lin, Chen Wang, Tianya Wang, Shucai Wang

**Affiliations:** 1 Laboratory of Plant Molecular Genetics & Crop Gene Editing, School of Life Sciences, Linyi University, Linyi 276000, China; 2 Key Laboratory of Molecular Epigenetics of MOE, Northeast Normal University, Changchun 130024, China

**Keywords:** AtbZIP62, transcription factor, abscisic acid, CRISPR/Cas9, Arabidopsis

## Abstract

The basic region/leucine zipper (bZIP) transcription factor AtbZIP62 is involved in the regulation of plant responses to abiotic stresses, including drought and salinity stresses, NO_3_ transport, and basal defense in Arabidopsis. It is unclear if it plays a role in regulating plant responses to abscisic acid (ABA), a phytohormone that can regulate plant abiotic stress responses via regulating downstream ABA-responsive genes. Using RT-PCR analysis, we found that the expression level of *AtbZIP62* was increased in response to exogenously applied ABA. Protoplast transfection assays show that AtbZIP62 is predominantly localized in the nucleus and functions as a transcription repressor. To examine the roles of AtbZIP62 in regulating ABA responses, we generated transgenic Arabidopsis plants overexpressing *AtbZIP62* and created gene-edited *atbzip62* mutants using CRISPR/Cas9. We found that in both ABA-regulated seed germination and cotyledon greening assays, the *35S:AtbZIP62* transgenic plants were hypersensitive, whereas *atbzip62* mutants were hyposensitive to ABA. To examine the functional mechanisms of AtbZIP62 in regulating ABA responses, we generated Arabidopsis transgenic plants overexpressing *35S:AtbZIP62-GR*, and performed transcriptome analysis to identify differentially expressed genes (DEGs) in the presence and absence of DEX, and found that DEGs are highly enriched in processes including response to abiotic stresses and response to ABA. Quantitative RT-PCR results further show that AtbZIP62 may regulate the expression of several ABA-responsive genes, including *USP, ABF2*, and *SnRK2.7*. In summary, our results show that *AtbZIP62* is an ABA-responsive gene, and AtbZIP62 acts as a transcription repressor to positively regulate ABA responses in Arabidopsis.

## 1. Introduction

Due to their sessile nature, plants are constantly challenged by biotic stresses, such as various pathogens and abiotic stresses, including drought and high salinity [1]. To survive, plants had to respond properly to environmental stresses. The phytohormone abscisic acid (ABA) plays a key role in regulating plant responses to abiotic stresses [2,3]. The regulation of plant responses to abiotic stresses by ABA is usually linked to osmotic stress that can promote ABA accumulation [4,5]. Whereas ABA signal transduction leads to the activation/suppression of stress-related genes, affecting plant responses to abiotic stresses [6,7].

Several ABA signaling key regulatory components have been identified, such as the ABA receptors pyrabactin resistance/PYR1 like/regulatory components of ABA receptors (PYR/PYL/RCAR), the type 2C protein phosphatases (PP2Cs), the protein kinases Snf1 (sucrose-non-fermentation 1)-related kinases subfamily 2 (SnRK2s), and the downstream ABA-responsive element binding protein/ABRE-binding factor/ABA INSENSITIVE 5 (ABF/AREB/ABI5)-type basic region leucine zipper (bZIP) transcription factors [6,7,8,9,10,11,12]. Signal transduction through the PYR/PYL/RCABR receptors, the PP2C phosphatases, and the SnRK2 kinases activate the ABF/AREB/ABI5-type bZIP transcription factors, resulting in the activation or repression of downstream ABA response genes, and thereby changes of plant responses to abiotic stresses [6,7,13,14,15,16].

In addition to the ABF/AREB/ABI5-type bZIP transcription factors, sever other transcription factors have also been reported to be involved in regulating ABA signaling. For example, APETALA2/ETHYLENE RESPONSIVE FACTOR (AP2/ERF), basic/helix-loop-helix (bHLH), Homeodomain-leucine zipper (HD-Zip), MADS-box, MYB, NAC, GARP (Golden2, ARR-B, Psr1), WRKY, AITR (ABA-induced transcription repressor) and the WDR (WD40-repeat) proteins [17,18,19,20].

The bZIP family transcription factors are characterized by a highly conserved bZIP domain and one of the biggest transcription factor families [12,21]. There are 75 bZIP transcription factors in Arabidopsis, 50 of which have not yet been functionally characterized [22]. Further comprehensive analyses identified two additional bZIP transcription factors in Arabidopsis [23]. It has been shown that bZIP transcription factors in plants mainly recognize ACGT core motif-containing cis-acting DNA elements, such as TACGTA (A-box), GACGTC (C-box), and CACGTG (G-box) [12,24,25]. The bZIP family proteins in Arabidopsis regulate different physiological processes in plants, including seed germination, floral growth and fertility, plant senescence, responses to abiotic stresses, and ABA signal transduction [26]. As examples, AtbZIP9, AtbZIP12 & 39, AtbZIP14/FD, AtbZIP18, AtbZIP29, AtbZIP30, AtbZIP34, and AtbZIP44 are all involved in regulating plant development [27,28,29,30,31]. In particular, leaf senescence, a controlled developmental process that has a significant impact on food crop productivity and nutritional content [32,33], is also regulated by several bZIP transcription factors, including AtbZIP1, AtbZIP36/ABF5, AtbZIP37/ABF3, AtbZIP38/ABF4/AREB2), AtbZIP41 and AtbZIP53 [32,34,35]. In addition, some bZIP transcription factors, such as AtbZIP56, are also involved in regulating plant metabolism [36]. So far, a number of bZIP transcription factors, such as AtbZIP1, AtbZIP17, AtbZIP24, AtbZIP35/ABF1, AtbZIP36/ABF2/AREB1, AtbZIP51, and AtbZIP62 have been found to regulate plant abiotic and biotic stress responses in Arabidopsis [37,38,39,40,41,42,43,44].

AtbZIP62 has been previously shown to regulate plant responses to abiotic stresses such as drought [45] and salinity [44]. AtbZIP62 also plays a role in regulating NO_3_ transport [46] and acts as a negative regulator of basal defense [47]. In this study, we show that AtbZIP62 is involved in regulating ABA response in Arabidopsis. We found that *AtbZIP62* is an ABA inducible gene, AtbZIP62 functions as a transcription repressor, and Arabidopsis plants overexpressing *AtbZIP62* are more sensitive, while *atbzip62* mutants are less sensitive to ABA.

## 2. Results

### 2.1. Expression of AtbZIP62 in Response to ABA and in Different Tissues and Organs

**Figure 1:**
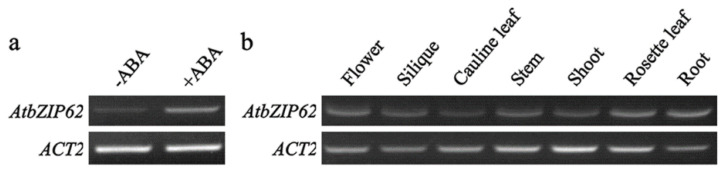
Expression of *AtbZIP62* in response to ABA treatment and in different tissues and organs. (**a**) Expression of *AtbZIP62* in response to ABA treatment. Col wild-type seedlings of10-day-old were treated for 4 h with 50 μM ABA, RNA was isolated and used for RT-PCR to examine the expression of *AtbZIP62*. *ACT2* was used as a control for RT-PCR. (**b**) Expression pattern of *AtbZIP62*. Different tissues and organs were collected from the Col wild-type Arabidopsis plants. RNA was isolated and used for RT-PCR to examine the expression of *AtbZIP62*. *ACT2* was used as a control gene for RT-PCR.

ABA regulates plant responses to abiotic stresses via activation/suppression of downstream genes [6,7]. Previous experiments have shown that the AtbZIP62 is involved in the regulation of plant responses to abiotic stresses such as drought and salinity [44,45]. To examine if ABA can regulate the expression of *AtbZIP62*, we compared the expression levels of *AtbZIP62* in Arabidopsis seedlings treated with ABA and control samples. RNA was extracted from Arabidopsis seedlings treated with ABA or solvent for 4 h, and the expression of *AtbZIP62* was determined using RT-PCR. As shown in Figure 1a, exogenous application of ABA significantly increased the expression level of *AtbZIP62* when compared to the mock-treated control.

We also examine the expression patterns of *AtbZIP62*. RNA was isolated from different tissues and organs collected from Col wild-type Arabidopsis plants and used for RT-PCR analysis. As shown in Figure 1b, the transcripts of *AtbZIP62* were detectable in all the tissues and organs examined with almost similar expression levels.

### 2.2. AtbZIP62 Is a Transcription Repressor

**Figure 2:**
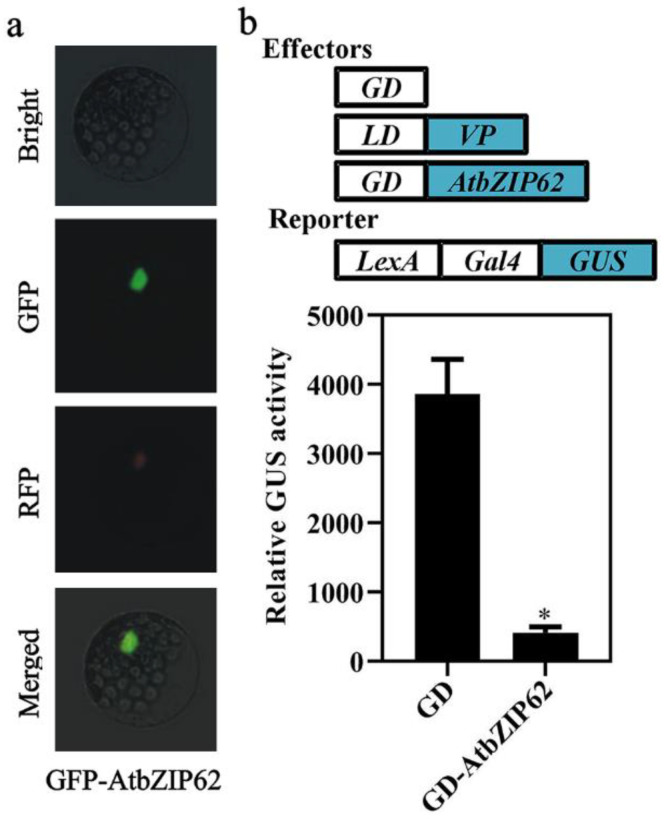
Subcellular localization and transcriptional activities of AtbZIP62. (**a**) Subcellular localization of AtbZIP62. Plasmids of the effector *GFP-AtbZIP62* and the nuclear marker gene *NLS-RFP* were co-transfected into Arabidopsis protoplasts isolated from leaves of the Col wild-type plants. The transfected protoplasts were incubated in the dark at room temperature for 20–22 h, and GFP and RFP fluorescence was observed and photographed under a confocal microscope. (**b**) Transcriptional activities of Arabidopsis AtbZIP62. Plasmids of the *GD-AtbZIP62* effector, the *LD-VP* activator, and the *LexA-Gal4:GUS* reporter constructs were co-transfected into Arabidopsis protoplasts isolated from the Col wild-type plants, the transfected protoplasts were incubated in darkness for 20–22 h, and then GUS activity was assayed by using a microplate reader. Co-transfection of the *GD* construct plasmids served as a control. Data represent the mean ± SD of three replicates. * significantly different from the GD control (student’s *t*-test, *p* < 0.001).

As AtbZIP62 is a transcription factor whose transcriptional activities have not been tested, we examined the transcription activities using Arabidopsis protoplast transfection. We first examined the subcellular localization of AtbZIP62. Plasmids of *GFP-AtbZIP62* and *NLS-RFP*, a nuclear marker gene construct, were co-transfected into Arabidopsis protoplasts, and the fluorescence of GFP and RFP were examined under a fluorescence microscope. As shown in Figure 2a, GFP fluorescence was predominantly observed in the nucleus.

After showing that AtbZIP62 is a nucleus protein, we examined its transcriptional activities using a protoplast transfection system successfully used to study transcription repressors [48]. Plasmids of the reporter construct *LexA-Gal4:GUS*, the activator construct *LD-VP*, and the effector construct *GD-AtbZIP62* or the control construct *GD* were co-transfected into Arabidopsis protoplasts. After overnight incubation of the transfected protoplasts in the dark, GUS activities were measured using a microplate reader. As shown in Figure 2b, co-transfection of *LD-VP* and *GD* activated the reporter gene, whereas co-transfection of *LD-VP* with *GD-AtbZIP62* resulted in repressed expression of the reporter gene, suggesting that AtbZIP62 functions as a transcription repressor.

### 2.3. Generation of the 35S:AtbZIP62 and the 35S:AtbZIP62-GR Transgenic Plants, and the Gene-Edited atbzip62 Mutants

To examine the functions of AtbZIP62, we generated plants overexpressing *AtbZIP62* under the control of *35S* promoter (*35S:AtbZIP62*) and CRISPR/Cas9 gene-edited *atbzip62* mutants.

**Figure 3:**
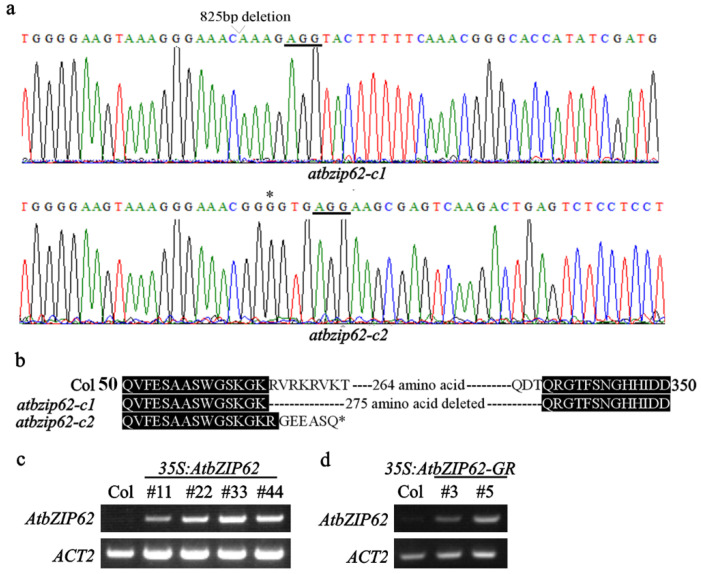
Generation of the transgenic overexpression lines and gene-edited mutants of *AtbZIP62.* (**a**) Editing status of *AtbZIP62* in the *atbzip62* mutants. DNA was isolated from leaves of T2 plants with normal flowering and used for RT-PCR amplification of *AtbZIP62* and sequencing. The underlines indicate the PAM sites, the open arrow end indicates the site where fragment deletion occurred, and the star indicates the single nucleotide inserted. (**b**) Amino acid alignment of AtbZIP62 in the Col wild type plant and the *atbzip62* mutants. The ORFs of *AtbZIP62* in the *atbzip62* mutants were identified using ORF finder (https://www.ncbi.nlm.nih.gov/orffinder, accessed on 9 December 2020), and corresponding amino acid sequences were aligned with the amino acid sequence of AtbZIP62. (**c**,**d**) Expression of *AtbZIP62* in the *35S:AtbZIP62* & *35S:AtbZIP62-GR* transgenic plants. Total RNA was isolated from seedlings of 10-day-old homozygous transgenic plants and used for RT-PCR analysis to examine the expression of *AtbZIP62*. *ACT2* was used as a control.

CRISPR/Cas9 mediated genome editing was used to generate the *atbzip62* mutants. Two target sites in the exon of *AtbZIP62* were selected, and the corresponding sequence was used to generate the *pHEE-FT-AtbZIP62* construct using the *pHEE-FT* vector [49]. The construct was transformed into the Col wild-type Arabidopsis plants. Editing status in early bolting T1 plants was examined, and Cas9-free homozygous mutants were isolated from T2 plants. Two independent homozygous mutants were chosen for further experiments. In the *atbzip62-c1* mutant, a deletion of 825 nucleotides fragment occurred in *AtbZIP62* (Figure 3a), resulting in a 275 amino acids deletion (Figure 3b). In the *atbzip62-c2* mutant, a single nucleotide was inserted in *AtbZIP62* (Figure 3a), leading to the substitution of a few amino acids and a premature stop in AtbZIP62 (Figure 3b).

The *35S:AtbZIP62* overexpression transgenic plants were generated by transforming the Col wild-type Arabidopsis plants with *pPZP211-35S:AtbZIP62*. In the T3 generation, homozygous plants were selected, and the expression level of *AtbZIP62* in homozygous transgenic plants was examined. The #22 and #33, two independent lines with similar expression levels of the *AtbZIP62* gene (Figure 3c), were selected and used for the following experiments. To examine genes that may be regulated by AtbZIP62, we generated *35S:AtbZIP62-GR* transgenic plants, in which the AtbZIP62 protein was fused with the glucocorticoid receptor (GR) (Figure 3d).

### 2.4. AtbZIP62 Positively Regulates ABA Response in Arabidopsis

**Figure 4:**
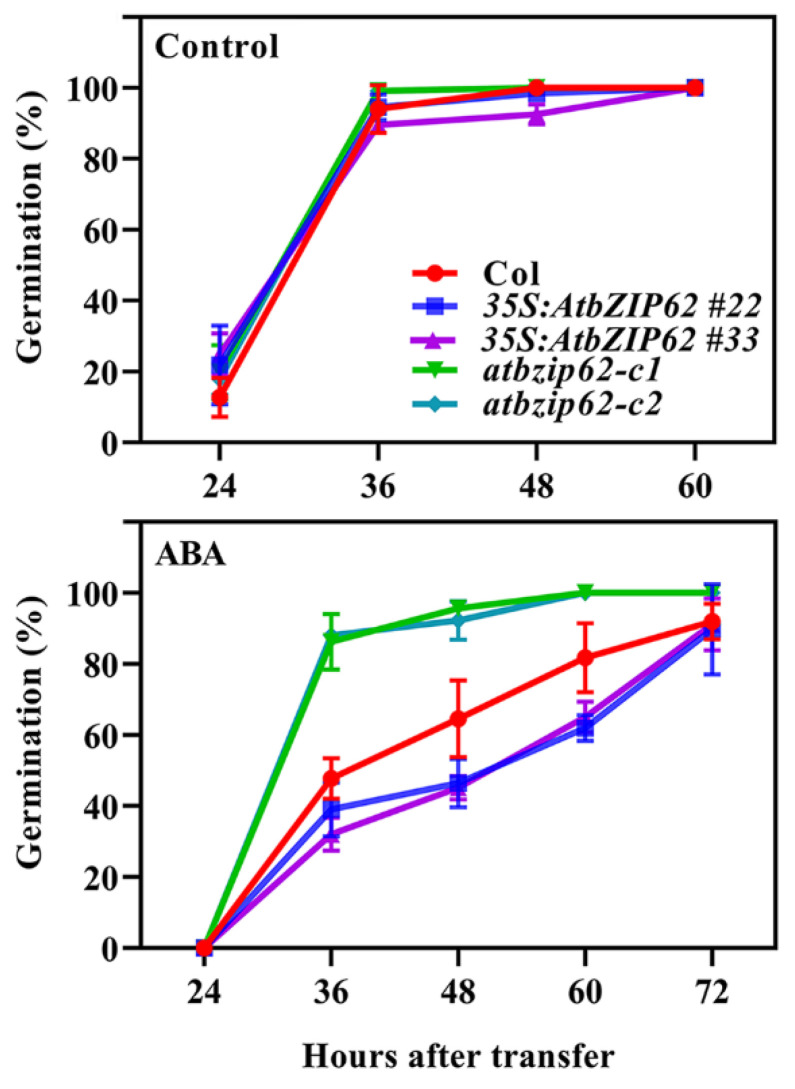
Effect of ABA on seed germination of the Col wild type, the *35S:AtbZIP62* transgenic plants, and the *atbzip62* mutants. Sterilized seeds were sown on 1/2 MS plates with or without 1 μM ABA, kept at 4 °C and in darkness for 2 days, and then transferred to a growth room. The number of germinated seeds was counted every 12 h after the transfer until all the seeds were germinated. The percentage of seed germination was then calculated. Data represent the mean ± SD of three replicates.

Having shown that *AtbZIP62* is an ABA-responsive gene (Figure 1a), we further explored the functions of AtbZIP62 in regulating ABA responses in Arabidopsis by using the overexpression transgenic plants and mutants generated. ABA-inhibited seed germination and cotyledon greening assays were used to examine ABA responses of the Col wild-type, the *35S:AtbZIP62* transgenic plants, and the *atbzip62* mutants. In the seed germination assays, we found that seed germination of different genotypes was similar in the absence of ABA, i.e., almost all the seeds germinated 36 h after the plates were transferred to the growth room (Figure 4). In the presence of ABA, however, lower germination rates were observed for seeds of the *35S:AtbZIP62* transgenic plants, whereas higher germination rates were observed for seeds of the *atbzip62* mutants at all the time points examined (Figure 4), indicating that ABA sensitive was reduced in the *atbzip62* mutants but increased in the *35S:AtbZIP62* transgenic plants.

**Figure 5:**
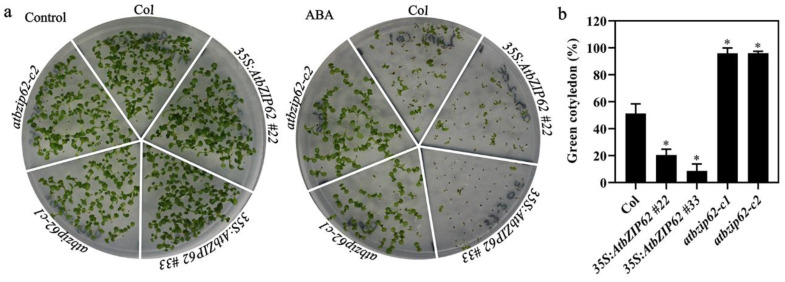
Effects of ABA on cotyledon greening of Col wild type, the *35S:AtbZIP62* transgenic plants and the *atbzip62* mutants. (**a**) Cotyledon greening of Col wild type, the *35S:AtbZIP62* transgenic plants, and the *atbzip62* mutants in the presence and absence of ABA. Sterilized seeds were sown on 1/2 MS plates in the presence or absence of 1 μM ABA. After being kept at 4 °C in darkness for 2 days, the plates were transferred to a growth room. Pictures were taken 8 days after the transfer. (**b**) Quantitative assays of cotyledon greening of the Col wild type, the *35S:AtbZIP62* transgenic plants, and the *atbzip62* mutants in the presence of 1 µM ABA. Seedlings with green cotyledons were counted 8 days after the transfer, and the percentage of green seedlings was calculated. Data represent means ± SD of three replicates. * significantly different from that of the Col wild type (student’s *t*-test, *p* < 0.005).

Similar results were obtained in the cotyledon greening assays. As shown in Figure 5a, in the presence of ABA, the numbers of green seedlings of the *35S:AtbZIP62* transgenic overexpression plants were lower than those of Col wild type plants, while that of the *atbzip62* mutant plants were clearly higher than those of Col wild type plants. Quantitative results show that at the present of ABA, the cotyledon greening rate for the Col wild type was ~50%, that for the *35S:AtbZIP62* transgenic plants was ~20%, whereas that for the *atbzip62* mutants was ~100% (Figure 5b). These results suggest that AtbZIP62 positively regulates ABA responses in Arabidopsis.

### 2.5. AtbZIP62 Regulated Genes Are Enriched in Response to ABA

To explore the functional mechanism of AtbZIP62 in regulating ABA response in Arabidopsis, we decided to investigate the downstream gene whose response to ABA may be regulated by AtbZIP62. We, therefore, generated the *35S:AtbZIP62-GR* transgenic plants (Figure 3d) and used them for transcriptome analysis.

In transgenic plants, the fusion proteins cannot enter the nucleus in normal conditions and are not functional, but they can enter the nucleus when treated with dexamethasone (DEX), and AtbZIP62 can regulate the expression of its downstream genes. We, therefore, performed comparative transcriptome analysis with 10-day-old seedlings of the *35S:AtbZIP62-GR* plants treated with 50 µM ABA and 50 µM ABA + 10 µM DEX for 4 h. Since AtbZIP62 functions as a transcriptional repressor (Figure 2b), we focus on the genes that were down-regulated in ABA + DEX-treated seedlings. 

**Figure 6:**
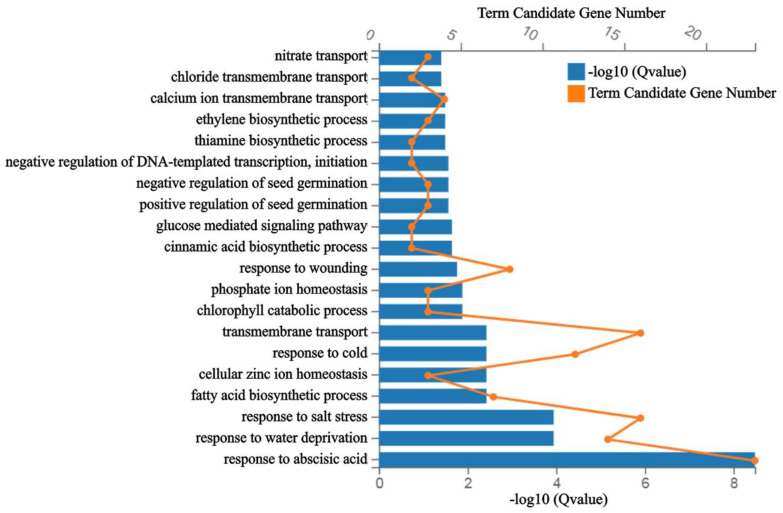
Functional categorization of the differentially expressed genes. Function categorization of the DEGs down-regulated in ABA + DEX treated *35S:AtbZIP62-GR* transgenic plant seedlings. DEGs down-regulated in ABA + DEX treated *35S:AtbZIP62-GR* transgenic plant seedlings were subject to Gene Ontology (GO) biological enrichment by using the pHyper function in R software and estimated by hypergeometric test. Adjusted *p*-values (−log10 or Q-values) depicting significant enrichment (q-value < 0.05).

A total of 231 different expressed genes (DEGs) were found down-regulated by ABA + DEX treatment in *35S:AtbZIP62-GR* transgenic plant seedlings (Table S1). Gene ontology (GO) term enrichment analysis reveals that these genes were enriched in several different processes such as transport, including nitrate transport, chloride transmembrane transport, calcium ion transmembrane transport, biosynthetic process (including ethylene biosynthetic, thiamine biosynthetic, and cinnamic acid biosynthetic), and fatty acid biosynthetic, regulation of DNA-template transcription, regulation of seed germination, glucose-mediated signaling pathway, and responses to environmental stimuli including wounding cold and salt stress. However, the genes were mostly enriched in response to ABA (Figure 6, Table S1).

### 2.6. AtbZIP62 Regulates ABA-Induced Expression of Some ABA and Abiotic Stress Response Regulator Genes

**Figure 7:**
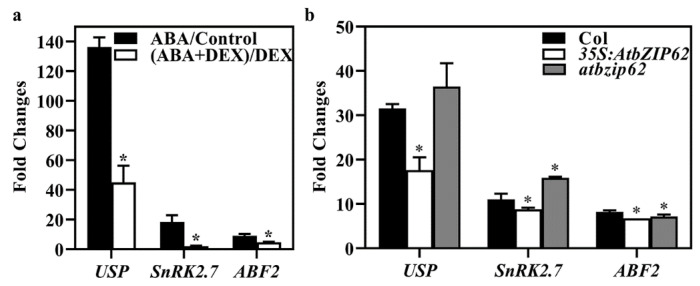
AtbZIP62 affects ABA-induced expression of *USP*, *SnRK2.7,* and *ABF2*. (**a**) ABA-induced expression of *USP*, *SnRK2.7,* and *ABF2* in ABA and ABA + DEX treated *35S:AtbZIP62-GR* transgenic plant seedlings. Ten-day-old seedlings of *35S:AtbZIP62-GR* transgenic plants were treated with 50 μM ABA in the presence and absence of 10 μM DEX for 4 h. RNA was then isolated and used for qRT-PCR analysis. *ACT2* was used as an inner control, and fold changes were calculated by comparing the expression levels of the corresponding genes in ABA-treated with control seedlings in the presence and absence of DEX, respectively. Data represent the mean ± SD of three replicates. * significantly different from ABA-treated seedlings (student’s *t*-test, *p* < 0.005). (**b**) ABA-induced expression of *USP*, *SnRK2.7,* and *ABF2* in the *35S:AtbZIP62* transgenic plant and *atbzip62* mutant seedlings. Seedlings of 10-days-old Col wild type, the *35S:AtbZIP62* transgenic plants, and the *ztbzip62* mutants were treated with 50 μM ABA for 4 h. RNA was then isolated and subjected to qRT-PCR analysis. *ACT2* was used as an inner control, and fold changes were calculated by comparing the expression levels of the corresponding genes in ABA-treated with control seedlings. Data represent the mean ± SD of three replicates. * significantly different from that in the Col wild-type seedlings (student’s *t*-test, *p* < 0.05).

Among the DEGs down-regulated in ABA + DEX treated *35S:AtbZIP62-GR* transgenic plant seedlings, several genes, including *USP*, *SnRK2.7,* and *ABF2,* have been well studied and are known to be related to ABA and abiotic stress responses. The expression of *USP*, a universal stress protein gene, is induced by ABA, and ABA sensitivity in the *atusp* mutant was increased, as indicated by seed germination assays [50]. *SnRK2.7* responds strongly to osmotic stress and ABA [51], whereas ABF2 is a master regulator of ABA signaling, and the expression of *ABF2* is induced by ABA [33]. We, therefore, examined their expression in ABA and ABA + DEX treated *35S:AtbZIP62-GR* transgenic plant seedlings using qRT-PCR. As shown in Figure 7a, ABA response of all three genes was reduced, consistent with the transcriptome data (Table S1). We also examined the ABA-induced expression of *USP*, *SnRK2.7,* and *ABF2* in the *35S:AtbZIP62* transgenic plant and the *atbzip62* mutant seedlings, and found that ABA-induced expression of all three genes was reduced in the *35S:AtbZIP62* transgenic plant seedlings (Figure 7b).

## 3. Discussion

The bZIP transcription factor family is one of the largest transcription factor plant families and is involved in the regulation of a wide range of biological activities, including plant growth, development, and responses to abiotic and biotic stresses [22]. So far, several bZIP transcription factors, especially those from group A, including bZIP36/ABF2, bZIP37/ABF3, and bZIP38/ABF4 have been shown to be involved in the regulation of plant abiotic stress responses via regulating ABA responses [15,52,53]. It has been previously shown that AtbZIP62, a group V bZIP transcription factor [54], regulates plant responses to abiotic stresses such as drought and salinity [44,45]. We provide evidence here that *AtbZIP62* is an ABA-responsive gene, and AtbZIP62 regulates ABA responses in Arabidopsis.

It has been shown that the expression of *AtbZIP36* and *AtbZIP38* genes were induced by high-salt and ABA treatments [55]. Our data indicate that the expression level of *AtbZIP62* is significantly increased in response to exogenous ABA treatment (Figure 1a). To understand the roles of the *AtbZIP62* gene in Arabidopsis, we analyzed the expression pattern of the *AtbZIP62* in different tissues and organs, and found that *AtbZIP62* expressed at almost similar levels in all the tissues and organs examined (Figure 1b). The results suggest that AtbZIP62 may not play a particular role in plant growth and development. Protoplast transfection assays indicated that AtbZIP62 is a nuclear protein (Figure 2a) and can reduce the expression of the reporter gene activated by a transcription activator, indicating that AtbZIP62 is a transcription repressor (Figure 2b). This observation is consistent with the observation that most bZIP proteins are exclusively localized in the nucleus to exert their function as transcription factors [56].

It has been shown that ABA is involved in regulating seed dormancy, seed germination, and seedling growth [57] and that abiotic stresses can induce ABA biosynthesis and trigger ABA-dependent signaling transduction [58]. So far, most bZIPs that bind to ABREs, such as ABI5, ABRE1/ABF2, ABRE2/ABF4, and ABF1, are reported to belong to group A in bZIP transcription factors in Arabidopsis, and their gene expression is strongly induced by ABA and abiotic stresses [59,60]. A previous study investigated that AREB1 and AREB2 are the most well-known bZIP transcription factors that act as fundamental components in regulating ABRE-dependent gene expression. Overexpression analysis reveals that *ABF2*, *ABF3*, *ABF4,* and *ABI5* transgenic plants are more sensitive to ABA during seed germination and later growth stage [61,62]. Our results suggest that group V bZIP transcript factor AtbZIP62 also positively regulates ABA response in Arabidopsis, as the transgenic plants overexpressing *AtbZIP62* showed a hypersensitivity, while the *atbzip62* mutant showed a hyposensitivity response to ABA in both seed germination and green cotyledon assays (Figure 4 and Figure 5a,b).

Previous research has revealed that Arabidopsis bZIPs play various roles in plant growth, environmental signaling, and stress response [26]. Transcriptomic analysis revealed that DEGs downregulated in ABA + DEX treated *35S:AtbZIP62-GR* transgenic plant seedlings enriched in biological processes such as response to abscisic acid, wounding, and abiotic stimulus (Figure 6, Table S1). ABA-responsive genes within these clusters, including *USP*, *SnR2.7,* and *ABF2*, whose expression levels in response to ABA are significantly reduced in the *35S:AtbZIP62-GR* transgenic plants treated with DEX (Figure 7a). Therefore, it can be speculated that *AtbZIP62* overexpression affected ABA-regulated gene expression, which, in turn, affected plant response to abiotic stresses, as reported previously [44,45]. In addition, the results shown in Figure 7b indicate that the expression of *USP*, *SnRK2.7,* and *ABF2* in response to ABA was affected in the *AtbZIP62* overexpress lines and the *atbzip62* mutants. Considering that *USP* and *ABF2* are regulated by phytohormones and involved in regulating seed germination in Arabidopsis [42,50], it is likely that AtbZIP62 regulates seed germination via regulating the expression of *USP and ABF2*.

## 4. Materials and Methods

### 4.1. Plant Materials and Growth Conditions

The Columbia-0 (Col) wild-type Arabidopsis (*Arabidopsis thaliana*) stored in our lab was used as a control for gene expression pattern and ABA sensitivity assays, for protoplasts isolation, and for plant transformation to generate transgenic overexpression plants and gene-edited mutants.

For ABA treatment, gene expression analysis, transcriptome analysis, seed germination, and cotyledon greening assays, seeds of the Col wild-type, the *AtbZIP62* and *AtbZIP62-GR* transgenic plants, and the *atbzip62* mutants were surface sterilized, rinsed with sterilized water, and then sowed on ½ MS (Murashige and Skoog) plates containing 1% sucrose and 0.6% agar. The plates were incubated in darkness at 4 °C for two days before being transferred to a growth room. For gene expression pattern assay, protoplast isolation, and plant transformation, the Col wild-type seeds were germinated and grown in soil pots in a growth room. The growth room conditions were set at 22 °C with a light density of ~120 μmol m^−2^ s ^−1^ under 16/8 h light/dark conditions.

### 4.2. DNA and RNA Isolation, RT-PCR, and Quantitative RT-PCR (qRT-PCR)

DNA and total RNA were extracted, and cDNA was synthesized as previously described [63,64]. In brief, the total RNA in the samples collected was isolated by using the EasyPure Plant RNA Kit (TransGene Biotech, Beijing, China), and cDNA was synthesized by using the EasyScript FirstStrand DNA Synthesis Super Mix (TransGen Biotech, Beijing, China) according to the manufacturer’s protocols. DNA was isolated using a DNA isolation buffer, as described previously [65].

For expression pattern assay of *AtbZIP62*, tissues and organs were collected from Col wild-type Arabidopsis plants grown in soil pots. To examine the expression of AtbZIP62 in response to ABA treatment, ABA-treated and control seedlings were collected. To examine the expression of *AtbZIP62* in the overexpression of transgenic plants, seedlings of homozygous transgenic plants were collected. The collected samples were frozen in liquid N_2_ immediately and stored at −80 °C for RNA isolation.

To examine gene editing status, DNA was extracted from the leaves of T1 or T2 transgenic plants. DNA isolated from T1 transgenic plants was used for PCR amplification of *AtbZIP62* genome sequences. The PCR products were isolated and sequenced, and the sequencing results were aligned with the wild-type *AtbZIP62* sequence to select the gene-edited T1 transgenic plants. DNA isolated from T2 progeny of the altered T1 plants was used for PCR amplification to isolate Cas9-free homozygous mutants. The primers used to amplify the *AtbZIP62* genomic sequence are *AtbZIP62-F* 5′-ATGGAGTTGGAGCCTATATCA-3′ and *AtbZIP62-R* 5′-TTATCCGACCTGCATCC-3′. The primers used for PCR amplification of Cas9 have been described previously [66].

The expression of *AtbZIP62* in response to ABA treatment, the expression pattern of *AtbZIP62* in different tissues of the Col wild-type plants, and the expression level of *AtbZIP62* in the overexpression transgenic plants were examined by using RT-PCR. The expression of ABA inducible genes, i.e., *USP*, *Snrk2.7,* and *ABF2,* was examined using qRT-PCR. *ACTIN2* (*ACT2*) was used as a control for RT-PCR and qRT-PCR. The qRT-PCR primer are, *USP*, 5′-GGAGACGGCTGCAAATAAGA-3′ and 5′-CCGGTTCGGCACTAATAACA-3′, *SnRK2*.7, 5′-GAAGATCCACGGAACATTAG-3′ and 5′-CAACACTCTGTCGACACTTC-3′, *ABF2*, 5′-TTGGGGAATGAGCCACCAGGAG-3′, and 5′-GACCCAAAATCTTTCCCTACAC-3′. The primers used for RT-PCR and quantitative RT-PCR (qRT-PCR) analysis of *ACT2* have been described previously [67].

### 4.3. Constructs

The reporters construct *LexA-Gal4:GUS*, the effector constructs *GD, LD-VP* and the *NLS-RFP* nuclear indicator construct for protoplast transfection were described previously [68,69,70].

To generate *GD-AtbZIP62* and *GFP-AtbZIP62* constructs, the full-length open reading frame (ORF) of *AtbZIP62* was RT-PCR amplified using RNA isolated from 10-day-old Col seedlings as described previously [63,64] and cloned into the *pUC19* vector under the control of the *CaMV 35S* promoter, and in frame with an N-terminal GD tag for transcriptional activity assay or a GFP tag for subcellular localization assay.

To generate *35S:AtbZIP62* constructs, the full-length ORF sequence of *AtbZIP62* was amplified and cloned into the *pUC19* vector with an in-frame N-terminal HA tag and under the control of the *35S* promoter [68,71]. To generate the *35S:AtbZIP62-GR* construct for plant transformation, the full-length ORF of *AtbZIP62* without stop codon was PCR amplified and cloned in frame with an N-terminal HA tag a C-terminal GR tag into *pUC19* vector. The *pUC19-35S:AtbZIP62* and *pUC19-35S:AtbZIP62-GR* constructs were digested with corresponding restriction enzymes and then cloned, respectively, into the binary vector *pPZP211* [72]. The primers (underlines indicate enzyme sites) used to amplify *AtbZIP62* were 5′-CAACATATGGAGTTGGAGCCTATATCA-3′ and 5′-CAAGAGCTCTTATCCGACCTGCATCC-3, the reverse primer used to amplify *AtbZIP62* without stop codon was 5′-CAAGAGCTCTCCGACCTGCATCCGAC-3′.

To generate *AtbZIP62* gene editing CRISPR/Cas9 constructs, potential target sequences were identified on CRISPRscan (http://www.crisprscan.org/?page=sequence, accessed on 1 September 2019) by scanning the exon sequences of *AtbZIP62* and then evaluated their specificity on Cas-OFFinder (http://www.rgenome.net/cas-offinder, accessed on 1 September 2019). The specific target sequences selected for editing *AtbZIP62* are 5′-GAAGTAAAGGGAAACGGGTG(AGG)-3′ and 5′- GGGCTTCAAGATACACAAAG(AGG)-3′. The target sequences were inserted into the previously described *pHEE-FT* vector [49]. The primers (underlines indicated the target sequences) used to generate *AtbZIP62* gene editing CRISPR/Cas9 construct were DT1-BsF 5′-ATATATGGTCTCGATTGAAGTAAAGGGAAACGGGTGGTT-3′ and DT1-F0 5′-TGAAGTAAAGGGAAACGGGTGGTTTTAGAGCTAGAAATAGC-3′, DT2-R0 5′-AACCTTTGTGTATCTTGAAGCCCAATCTCTTAGTCGACTCTAC-3′ and DT2-BsR 5′-ATTATTGGTCTCGAAACCTTTGTGTATCTTGAAGCCCAA-3′. U626-IDF and U629-IDR, the primers used for colony PCR and sequencing the generated CRISPR/Cas9 construct, have been described previously [73].

### 4.4. Plasmid DNA Isolation, Protoplasts Isolation, Transfection, GFP and RFP Fluorescence Observation, and GUS Activity Assays

Plasmid isolation, Arabidopsis protoplast isolation, transfection and incubation, GFP inflorescence observation, and GUS activity assays were performed as described previously [63,74,75,76]. In brief, plasmid DNA including *GFP-AtbZIP62, NLS-RFP, GD, GD-AtbZIP62, LD-VP,* and *LexA-Gal4:GUS* were isolated by using a GoldHi EndoFree PlasmidMaxi Kit (CWBIO, Taizhou, China) as instructed by the manufacturer, and protoplasts were isolated from rosette leaves collected from 3 to 4-week-old Col wild type plants.

For the subcellular localization assay, plasmids of the effector construct *GFP-AtbZIP62* and nuclear indicator construct *NLS-RFP* were co-transfected into the Arabidopsis protoplasts. For the transcriptional activity assays, the reporter plasmid construct *LexA-Gal4:GUS*, the activator construct *LD-VP,* and the effector construct *GD-AtbZIP62,* or the control construct *GD,* were co-transfected into Arabidopsis protoplasts. The transfected Arabidopsis protoplasts were incubated at room temperature for 18–22 h in darkness. Fluorescence of GFP and RFP was observed under a fluorescent microscope (Olympus, Tokyo, Japan), and GUS activities were measured using a Synergy HT fluorescence microplate reader (BioTEK, Vermont, USA).

### 4.5. Plant Transformation and Transgenic Plants Selection

Col wild-type plants about 5-week-old and with several mature flowers on the main inflorescence stems were used for transformation by using the floral dip method [77]. The plants were transformed with the *35S:AtbZIP62*, the *35S:AtbZIP62-GR*, and the CRISPR/Cas9 constructs via *GV3101* agrobacterium cells to obtain transgenic overexpression plants, as well as gene-edited mutants for gene *AtbZIP62*.

To select transgenic plants, T1 seeds were collected from the transformed plants and plated on ½ MS plates containing 100 μg/mL carbenicillin and 50 μg/mL kanamycin, and the transgenic plants selected were transplanted to soil pots and grown in a growth room. For *35S:AtbZIP62* and *35S:AtbZIP62-GR* overexpression plants, multiple homozygous lines were obtained, and the ones with high expression levels of *AtbZIP62* were used for the experiments.

The *atbzip62* Cas9-free gene-edited mutants were obtained by following the procedure described previously [49]. Briefly, the editing status of the *AtbZIP62* gene in T1 plants with early flowering phenotypes was examined. T2 seeds were collected from gene-edited T1 plants and sowed directly into soil pots, and the editing status of the *AtbZIP62* gene in the normal flowering (*Cas9*-free) was examined to identify homozygous mutants. Cas9-free status was further confirmed by amplifying the *Cas9* fragment in the homozygous mutants.

### 4.6. ABA Treatment

To examine the expression of *AtbZIP62* in response to ABA treatment, seedlings of 10-day-old Col wild-type plants were transferred to a 50 mL falcon tube containing 50 µM ABA and shaken in the dark for 4 h. To examine the expression of *USP, SnRK2.7,* and *ABF2* in Col wild-type, the *35S:AtbZIP62* transgenic plants and the *atbzip62* mutant, seedlings of 10-day-old Col wild type, the *35S:AtbZIP62* transgenic plants and the *atbzip62* mutants were treated with 50 µM ABA for 4 h.

### 4.7. Seed Germination and Cotyledon Greening Assays

For phenotypic analysis, seeds were germinated and grown on ½ MS supplemented with or without 1 μm ABA. Seed germination was judged based on the emergence of an embryonic axis protrusion. Cotyledon greening was judged based on observing cotyledon expansion and turning green. Three independent experiments were carried out, and similar results were obtained.

### 4.8. Transcriptome Analysis

For transcriptome analysis, 10-day-old seedlings of the *35S:AtbZIP62-GR* transgenic plants were transferred to a falcon tube and treated for 4 h with 50 µM ABA or 50 µM ABA + 10 µM DEX, frozen in liquid N_2_ and sent to the company, Beijing Genomics Institute for transcriptome sequencing and analysis.

Down-regulated DEGs in ABA + DEX treated vs. ABA treated seedlings were subject to functional Gene Ontology (GO) under the term of biological process. The pHyper function in R software was used and estimated by hypergeometric test for the gene ontology (GO) annotation enrichment of DEGs analysis. GO terms with a Q value (corrected *p*-value) equal to 0.05 were considered significantly enriched.

## 5. Conclusions

Our results in this study show that *AtbZIP62* is an ABA-responsive gene. AtbZIP62 functions as a transcription repressor and positively regulates ABA responses in Arabidopsis, possibly via regulating the expression of some ABA signaling regulator genes.

## Data Availability

All data are presented in the manuscript and the supplementary material.

## Supplementary Materials

The following supporting information can be downloaded at: https://www.mdpi.com/article/10.3390/plants11223037/s1, Table S1: Genes downregulated in the ABA + DEX group in the *35S:AtbZIP62-GR* transgenic plants.
